# Chronic gvhd dictionary—eurograft cost action initiative consensus report

**DOI:** 10.1038/s41409-022-01837-w

**Published:** 2022-10-13

**Authors:** Ana Zelić Kerep, Atillio Olivieri, Helene Schoemans, Anita Lawitschka, Jörg Halter, Drazen Pulanic, Anne Dickinson, Hildegard T. Greinix, Steven Z. Pavletic, Kirk R. Schultz, Stephanie J. Lee, Daniel Wolff

**Affiliations:** 1 Division of Hematology, Department of Internal Medicine, University Hospital Center Zagreb, Zagreb, Croatia; 2 Department of Clinical and Molecular Sciences, University of Ancona, Ancona, Italy; 3 Department of Hematology, University Hospital Leuven, Leuven, Belgium; 4 Deparment of Public Health and Primary Care, ACCENT VV, KU Leuven—University of Leuven, Leuven, Belgium; 5 Stem Cell Transplant Unit, St. Anna Children’s Hospital, Medical University Vienna, Vienna, Austria; 6 Division of Hematology, University Hospital Basel, Basel, Switzerland; 7 University of Zagreb School of Medicine, Zagreb, Croatia; 8 Hematological Sciences, Institute of Cellular Medicine, Newcastle University, Newcastle, UK; 9 Division of Hematology, Medical University Graz, Graz, Austria; 10 Immune Deficiency Cellular Therapy Program, Center for Cancer Research, National Cancer Institute, National Institutes of Health, Bethesda, MD USA; 11 Division of Hematology/Oncology/BMT, British Columbia Children’s Hospital Research Institute, Vancouver, BC Canada; 12 Fred Hutchinson Cancer Center, Seattle, WA USA; 13 Department of Internal Medicine III, University of Regensburg, Regensburg, Germany

**Keywords:** Diagnosis, Epidemiology

## Abstract

Chronic graft versus host disease (cGVHD) affects patients after allogeneic hematopoietic stem cell transplantation (alloHSCT). This orphan disease poses a challenge for clinicians and researchers. The purpose of the cGVHD Dictionary is to provide a standardized structure for cGVHD databases on an international level, reconciling differences in data retrieval and facilitate database merging. It is derived from several consensus meetings of the EUROGRAFT consortium (European Cooperation in Science and Technology—COST Action CA17138) followed by a consensus process involving European Society for Blood and Marrow Transplantation (EBMT), US GvHD consortium and Center for International Bone Marrow Transplant Registry (CIBMTR). Databases used for the dictionary were: the National Institutes of Health (NIH) database, the Center for International Blood and Marrow Transplant Research, Applying Biomarkers to Minimize Long Term Effects of Childhood/Adolescent Cancer Treatment - Pediatric Blood and Marrow Transplant Consortium database, EBMT registry, the German-Austrian-Swiss GvHD registry, Italian Blood and Marrow Transplantation Society registry and Regensburg-Göttingen-Newcastle HSCT dataset. A four-part cGVHD Dictionary was formed based on the databases, consensus, and evidence in the literature. The Dictionary is divided into: (1) Patient characteristics, (2) Transplant characteristics, (3) cGVHD characteristics and (4) patient-reported quality of life, symptom burden and functional indicators.

## Introduction

Chronic graft versus host disease (cGVHD) is a serious complication of allogeneic hematopoietic stem cell transplantation (alloHSCT), a debilitating condition prompting long-term immunosuppression and increased mortality risk [1, 2]. This orphan disease poses a significant challenge for both clinicians and researchers, requiring continuous collaborative efforts. For instance, real world data (RWD) collection is routinely used in HSCT to better understand the impact of current practice on clinical outcomes like the registry of the European Society for Blood and Marrow Transplantation (EBMT) and the Center for International Bone Marrow Transplant Registry (CIBMTR). RWD has the potential of complementing research results derived from clinical trials to allow reaching more generalizable conclusions. RWD take into account larger populations and different geographical, cultural and socio-economic settings. However, international guidelines require data collection to be based on a number of basic quality principles, including the need for consistent definitions of terms used [3].

Historically, cGVHD was categorized as limited or extensive based on the Seattle criteria, nevertheless, as the field progressed the criteria became more specific [4]. As diagnostic and prognostic scoring tools evolved [5], the need for a standardized approach to diagnose and determine severity of cGVHD was recognized in 2005 when the National Institutes of Health (NIH) cGVHD Consensus recommended criteria for diagnosis and staging, revised in 2014 [6, 7]. However, there are still discrepancies in reporting among clinicians and researchers, due in part to the slight differences between the two sets of NIH recommendations [8] and implementation challenges linked to the lack of knowledge, lack of time and confidence of healtcare professionals in applying them [9, 10]. In fact, a large proportion of healthcare professionals have been shown to struggle with cGVHD recognition and evaluation [11**–**13]. Inconsistencies in collecting and reporting data pose an impediment for data interpretation and comparison [14]. Several electronic tools [12, 14, 15] are now available to support GHVD documentation. For instance, the eGVHD app (www.uzleuven.be/egvhd), which has shown promising results in improving reliable GVHD evaluation performed by healthcare professionals [13], was based on the EBMT-NIH-CIBMTR task force position statement on standardized terminology and guidance for graft-versus-host disease assessment [12]. This statement clarified many issues in the cGVHD field, but also emphasized the difficulties in adherence to a common set of criteria, even in the post-NIH cGVHD Consensus era [10].

A common cGVHD Dictionary is therefore an essential tool for effective prospective international collection of data, preventing discrepancies and differences in reporting. The main purpose of the cGVHD Dictionary is to facilitate the setup of cGVHD databases and to provide clear definitions for each variable, corroborated by relevant literature.

## Methods

The process of creating the cGVHD Dictionary was initiated by the EUROGRAFT COST action. COST (European Cooperation in Science and Technology, https://www.cost.eu/) is a funding organization for research and innovation networks. COST Actions are networks dedicated to scientific collaboration, open to scientists in all career stages, based on a 4-year program. EUROGRAFT is a COST Action (https://gvhd.eu/; CA17138) which has brought together cGVHD experts and scientists across Europe-since August 2018. This network aims to coordinate research and build a collaborative network for cGVHD research. At the first COST cGVHD meeting in Zagreb (held from 7th to 8th November 2018) a need for a comprehensive and uniform cGVHD Dictionary was recognized and thus, became one of the objectives of the Eurograft COST action. The reference databases used (variables and definitions of items for databases were shared direclty among the authors, and supplemented by published manuscripts based on databases including the NIH database, the CIBMTR (Center for International Blood and Marrow Transplant Research) database, ABLE (Applying Biomarkers to Minimize Long Term Effects of Childhood/Adolescent Cancer Treatment—Pediatric Blood and Marrow Transplant Consortium), the EBMT registry, the Italian Blood and Marrow Transplantation Society (GITMO) database, the German-Austrian-Swiss GvHD registry and the joint Regensburg-Göttingen-Newcastle joint HSCT dataset.

All listed variables were carefully selected based on available literature and expert consensus. To facilitate implementation all variables were fully described and categorized as being either mandatory or recommended. Mandatory items are necessary for a database/registry and are fundamental pieces of information, which should be considered for data analysis. This recommendation is evidence-based with regard to the impact of relevant endpoints in cGvHD research. Recommended items are those not essential for a database, but can add value to research and should be included if they are available and feasible or required for specific endpoints.

## Results

The most important items of the dictionary are shown in Table 1, with the complete dictionary being available in the Supplementary Material. For each variable, we suggested the format (dropdown menus, checkboxes, etc) to minimize the possibility of error.

**Table 1:** Excerpt from the full-version of the cGVHD dictionary.

*ITEM*	*DEFINITION*	*CATEGORY*
*cGVHD category*	cGVHD diagnosed according to the NIH diagnosis and staging criteria. All items in the NIH Staging Criteria Form should be included. Undefined other cGVHD—atypical signs and symptoms of alloreactivity falling outside the NIH 2014 diagnostic criteria. We recommend that all manifestations treated as cGVHD are documented. Manifestations possibly connected to cGVHD should be documented, even if they are not treated as such.	Mandatory
*Specific undefined other cGVHD manifestations*	Any immune-mediated event in the context of cGVHD should be documented. *Full list provided in the* Supplemental material	Mandatory
*Diagnosis of cGVHD*	Diagnostic criteria present [7]. Distinctive symptoms present. Biopsy (no evidence of cGVHD = 1/possible or likely cGVHD = 2/not done = 3) Document the specific symptom or sign leading to diagnosis (free field).	Mandatory

The dictionary is comprised of four sections: [1] patient characteristics [2], transplant characteristics [3], cGVHD variables, and [4] patient-reported quality of life, symptom burden and functional indicators. In addition, in the Supplementary Material we provide a module proposed by the NIH Consensus Project Task Force document [15], designed specifically for atypical cGVHD manifestations.

Patient characteristics are a set of demographic variables necessary for a cGVHD database, which includes for instance, date of cGVHD onset, race, which is important for studies on genetic disparities and alloHSCT outcomes and consanguinity of parents to be collected in pediatric (patients under 18 years of age) databases. In the patient characteristics section, date of birth (year and 15th of the birth month), age at the time of cGVHD diagnosis, age at transplant as well as transplant date, age at the time of enrollment into the database, and date of enrollment are considered to be equally important for a cGVHD patient database.

The date of cGVHD onset is a variable found commonly in various databases. Based on expert consensus, we recommend specifying what the clinician considers to be the onset; whether it is the onset of symptoms, biopsy confirmation or the initiation of systemic treatment. The scientific value of this approach is yet to be determined.

We recommend using actual dates (date of birth, date of transplantation, date of onset) to allow for a precise calculation of the kinetics of GVHD. In settings where the General Data Protection Regulation 2016/679 is applicable, it might be necessary to use only the year and 15th of the month. Time intervals should ideally be calculated automatically, and not inserted by hand, to minimize errors.

Transplant characteristics are an important section of the Dictionary. Mandatory items include disease and disease status prompting alloHSCT, date of transplant, donor information and conditioning. Some of these variables are of prognostic value, for example, shorter time from transplantation to cGVHD diagnosis is an adverse risk factor for non-relapse mortality and overall survival [16], as well as advanced disease at transplantation [17]. In that context, these variables can be of value for future reevaluation and analyses. Other items are recommended and are of value in studies with specific scientific questions pertaining to them.

The section of cGVHD items encompasses the NIH cGVHD staging criteria based on published guidelines [7]. The diagnosis of cGVHD should be captured as follows: diagnostic criteria present, or distinctive symptoms with positive biopsy, or distinctive symptoms without a positive biopsy. This should help evaluate the portion of patients with distinctive symptoms treated for cGVHD without histological confirmation. A special category for non-NIH defined manifestations caused by immunologically mediated host-reactivity should be incorporated into the database, particularly if their identification resulted in the initiation of immunosuppressive agents [18]. Manifestations not prompting treatment but considered to be associated with cGVHD are not considered mandatory. Classifying cGVHD as “NIH-defined” and “undefined other cGVHD” will allow to improve our understanding of the debilitating effect of alloreactivity on virtually all organs, including those less frequently affected. The pediatric ABLE trial demonstrated similar cellular and plasma biomarker patterns, regardless of whether patients presented with strictly NIH-defined manifestations of cGVHD versus other manifestations considered by clinicians to be induced by host-directed immunity [19]. Furthermore, any impairment unequivocally caused by non-cGVHD causes, should be documented as such, as recommended by the NIH criteria and NCCN guidelines [7].

The symptoms leading to cGVHD diagnosis also help clarify the natural history of the disease. A significant proportion of patients in clinical routine are treated for distinctive symptoms without histopathological confirmation or other associated symptoms, so capturing those symptoms will enrich our knowledge of the clinical heterogeneity of cGVHD, in line with the recent NIH 2020 conference advocating for early recognition of GVHD.

Even though the NIH staging criteria allow lung staging based on symptoms, we suggest that such practice should be avoided, and lung function tests should be performed in all patients. Also, we recommend recording the details of chest CT imaging, if performed, with an emphasis on documenting radiological findings [7].

As for liver manifestations, actual liver enzyme and bilirubin levels are valuable to distinguish between the distinct hepatitic and cholestatic subform of GvHD. This is of special importance as cholestatic abnormalities are regarded as a common symptom of acute and chronic GVHD and lead to the classification of overlap syndrome [20, 21] and biology based classification of the subtypes requires prior detailed documentation. For all organs, NIH scores should be documented individually, but documenting each specific organ manifestation is not mandatory, but recommended.

Finally, at the current stage, we consider that functional capacity patient-reported outcomes are recommended, depending on the purpose of data collection [22**–**24]. The use of properly validated tools is highly recommended, however, the tools listed in the fourth part of the Dictionary are non-exclusive examples and many other measures are available [24].

## Discussion and conclusion

Chronic GVHD has been the focus of notable research efforts, with significant progress in identification of pathophysiology and new treatment options. Nonetheless, considerable challenges remain, requiring new and innovative solutions in the field of cGVHD. One severe limitation is the relatively small number of patients, thus joint analyses of carefully documented clinical data is a high priority. However, the intricacies of clinical categorization of patients pose a significant burden. Differences in collecting cGVHD data make it difficult to compare data between registries, as well as merge databases. The ever-change in prognostic and staging systems also affects data collection consistency [5]. This work expands on the EBMT-NIH-CIBMTR Taskforce report [12], endorsed by the EUROGRAFT COST Action panel of experts, and it is based on both available scientific evidence and the combined knowledge and clinical experience of clinicians and researchers. However, it differs from the CIBMTR and EBMT current databases since it is more clinican-oriented, and gives clear guidelines on which items are mandatory and can be done during routine clinical practice and which are recommended, i.e., more exploratory measures. The concept of such a compendium of variables necessary for comprehensive cGVHD research has the potential to facilitate future research on variables driving the disease and its prognosis. Once published, this Dictionary can be used for cGVHD databases by all interested researchers and clinical centers providing care for cGVHD patients. Finally, combined analyses with higher patient numbers may enable new techniques like machine learning to identify variables driving the course of disease which in turn may result in reduction of documentation load and may permit patient tailored treatment approaches [25].

## Supplementary information

cGVHD Dictionary

## Data Availability

There is no relevant data pertaining to this paper. All relevant infomation can be found in the Supplementary material.
