# Supplementary material for: Chronic gvhd dictionary—eurograft cost action initiative consensus report
Source: Bone Marrow Transplant. 2022 Oct 13;58(1):68–71. doi: 10.1038/s41409-022-01837-w (PMC9812761; doi:10.1038/s41409-022-01837-w)
Supplement: Supplementary file 1 — cGVHD Dictionary [file 41409_2022_1837_MOESM1_ESM.docx]

**CHRONIC GRAFT-VERSUS-HOST DISEASE DICTIONARY – a standardized compendium for cGVHD databases**

**General comments about data collection:**

1. **All fields should be empty at baseline.**
2. **All fields recording information require to differentiate between the issue/condition being ‘present’, ‘absent’ or ‘not evaluated’.**
3. **‘Free fields’ are difficult to analyze in bulk and should be avoided.**
4. **Some fields may contain sensitive issues in centers subject to GDPR and may require adaption. These are identified with an asterix.**
5. **Fields categorized as checkbox style should include the “not evaluated” option.**
6. **Patient characteristics**

| *ITEM (type of field, suggested field name)* | *DEFINITION and suggested coding categories* | *CATEGORY* | *COMMENTARY* |
| --- | --- | --- | --- |
| *Patient ID (free field, ID)* | **Assigned by the center** | **Mandatory** | **UPN + EBMT center number in Europe, UPN and CIBMTR center code in the US. This information may differ depending on the clinical center and is subject to change over time. This should not allow patient identification if the center is located in a GDPR-subject region.** |
| *Gender (categorical field, ptsex)* | **Male=1/female=2/transgender=3** | **Mandatory** | **Male recipients of female donors have a higher risk of cGVHD (1)(2). For transgender individuals genetics and hormonal status should be captured.** |
| **Age at alloHSCT and cGVHD diagnosis-(calculated field. agealloHSCT, agedx), date of birth (date field – bdate)* | **Date of birth (use year and 15^th^of the birth month). Age at alloHSCT and at the time of cGVHD diagnosis can then be calculated fields.** | **Mandatory** | **Age is a possible risk factor for non-relapse mortality after HSCT (1).** |
| *Date of cGVHD onset (date field – datedx, consider checkboxes: symptoms versus biopsy confirmation versus the initiation of treatment)* | **The database handbook should specify whether the date of cGVHD onset is the date of the first symptoms, the first biopsy, or the initiation of treatment.** | **Mandatory** | **It is recommended to document the first symptoms diagnosed clinically or/and by histology (whichever comes first).** |
| *Time intervals (calculation fields).*  *Date field for date of enrollment to the database – enrolldate.* | **Separate fields for months or days from alloHSCT to cGVHD diagnosis (alloHSCTdx), and time from cGVHD diagnosis to database enrollment (Dxenroll), and time from transplant to enrollment (alloHSCTenroll) in the database and every subsequent evaluation time-point, if patients are being followed longitudinally. In that case, the survival data should be recorded.** | **Mandatory** | **Calculated from date of first cGVHD onset. Additional alloHSCTs and related events should be documented separately.** |
| *Race/ethnicity*  *(categorical field, race)* | **As applicable in a specific population, to determine genetic background.** | **Recommended** | **According to the work of Madbouly et al. (3), these self-identified race/ethnicities correlate well with genetic markers: Caucasian, African-American, Asian/Pacific Islander, Hispanic, Native American, Other/multiple/declined/unknown**  **Race should be preferentially documented rather than ethnicity (or both) since race depicts genetic heritage and ethnicity usually refers to the way a person feels in a cultural and socioeconomic context (4). It could be of benefit in research regarding genetic disparities and alloHSCT outcomes, especially in combination with genetic research (3). Minor histocompatibility antigens also may differ between different racial/ethnic populations (5).** |
| *Center of alloHSCT*  *(free field, center)* |  | **Recommended** | **EBMT and/or CIBMTR center code** |
| *Karnofsky performance score (numerical field, KPS)* | **KPS or ECOG (Lansky for pediatric patients) at cGVHD diagnosis. (standard clinical center practice).** | **Recommended** | **KPS >80% is a favorable prognostic factor for NRM and OS (1,6).** |

1. **Transplant characteristics**

| *ITEM* | *DEFINITION* | *CATEGORY* | *COMMENTARY* |
| --- | --- | --- | --- |
| **Date of transplant (date field, datealloHSCT)* | **Date of alloHSCT – day/month/year (use year and 15th of the alloHSCT month to accommodate GDPR principles for EU countries which need to take this into account).** | **Mandatory** | **Time to cGVHD diagnosis is a risk factor for NRM and OS (7–9).** |
| *Relapse risk (categorical field, relapserisk)* | **Coding according to EBMT risk score 1-3 (10) or CIBMTR Disease Risk Index (11).** | **Mandatory** | **Advanced disease at alloHSCT is an adverse OS factor (12).** |
| *Underlying disease (categorical field. disease)* | **Main disease indication for alloHSCT** | **Mandatory** | **Coding is up to the center, EBMT and CIBMTR coding/grouping are encouraged.** |
| *Type of conditioning (categorical field, cond)* | **Myeloablative vs. Non-myeloablative vs Reduced-intensity**  **The term myeloablation refers to the administration of total body irradiation (TBI) and/or alkylating agents, at doses that will not allow autologous hematologic recovery: TBI >5 Gy single dose or >8 Gy if fractionated, Bu > 8 mg/kg PO or IV equivalent.**  **Non-myeloablative regimens consist of: TBI ≤ 2 Gy± purine analog, Flu + Cy ± ATG, Flu +AraC + Ida, Cladribine + AraC, Total Lymphoid Irradiation + ATG.**  **Regimens like Mel + Flu or Treo + Flu) that do not fit in either of these two categories are considered reduced-intensity conditioning (13).** | **Mandatory** | **Some studies suggest a higher incidence of cGVHD associated with myeloablative regimens (14).**  **We suggest to use the 'Champlin Criteria' which define reduced-intensity regimens as those that do not require stem cell support for hematopoietic recovery and that results in low non-hematologic toxicity and mixed donor-recipient chimerism in a substantial proportion of patients in the early post-transplant period (around day +30) (15).** |
| *TBI (categorical field - TBI + numerical field)* | **Options: 'yes','no' and if yes provide the total dose in Gy.** | **Recommended** | **TBI is a potential risk factor for sclerotic cGVHD manifestations (16).** |
| *Donor age (numerical field, donorage)* | **Age at the time of donation in years (data protection).** | **Recommended** | **Older donors pose a risk for cGVHD and NRM (1,17,18)** |
| *CMV status of the patient at the time of alloHSCT (categorical field)* | ***Suggested coding categories: positive (*positive IgG or equivocal, regardless of IgM status), negative (negative IgG and IgM)** | **Recommended** | **Pre-transplant cytomegalovirus (CMV) serostatus is an important determinant of CMV reactivation after allogeneic hematopoietic stem cell transplantation and impacts cGVHD severity (19)(20).** |
| *CMV status of donor (categorical field, CMVIgG, CMVIgM)* | ***Suggested coding categories: positive (*positive IgG or equivocal, regardless of IgM status), negative (negative IgG and IgM)** | **Recommended** | **A CMV seropositive unrelated donor for a CMV seronegative patient has a negative influence on overall survival, relapse-free survival and non-relapse mortality. Donor anti-CMV-IgG positivity is beneficial in regards to short-term immunological recovery (21,22)** |
| *Donor relationship (categorical field, donorrel)* | **Suggested coding categories based on CIBMTR (23): syngeneic (monozygotic twins), HLA-matched relative, HLA-identical sibling (non-monozygotic twins, siblings with identical HLA, excludes half-siblings), HLA-mismatched relative (includes half siblings), HLA-matched unrelated donor (10/10 matched on a molecular level), HLA-mismatched unrelated donor, haploidentical** | **Mandatory** | **Studies suggest a higher incidence of cGVHD in unrelated donor alloHSCTs** (**1,24,25)**. |
| *Donor gender (categorical field. donorsex)* | ***Suggested coding categories:* Male, female, transgender** | **Mandatory** | **Male recipients of female donors have a higher risk of cGVHD (1–3,26). For transgender people genetics and hormonal status should be captured as an exploratory effort. The scientific value of this may be determined in future analyses** |
| *Female donor parity (numerical field. parity)* | **The number of times the female donor was pregnant before stem-cell donation, including live births, miscarriages and abortions.** | **Recommended** | **Risk factor for cGVHD, probably due to alloimmunization (26).** |
| *Gender disparity (calculated field, genddisp)* | ***Suggested coding categories: Yes/No*** | **Mandatory** | **Should be automatically calculated based on recipient and donor gender** |
| *HLA mismatch (categorical field. HLAmis)* | **Document the number of antigens/alleles analyzed (10/10 or 12/12), Capture the degree of mismatch, where the mismatch is if available to the best possible level of detail, dependent on the method of typing (low- or high-resolution typing).** | **Recommended** | **Non-relapse mortality is associated with patient-donor HLA mismatch (26). Preferentially provide HLA-typing results.** |
| *Blood type (categorical field, bloodtype, calculated for mismatch)* | **Blood type of donor and recipient should be captured (27).** | **Recommended** | **Separate columns for patient and donor blood group and Rh factor.** |
| *Cell source and number of CD34+/kg infused. (categorical field, cellsour).* | ***Suggested coding categories:* Bone marrow=1/PBSC=2/ cord blood=3/G-CSF primed bone marrow=4** | **Mandatory** | **Stem cell source is an important factor for cGVHD and immune reconstitution (28,29). PBSC is associated with prolonged use of immunosuppressive therapy and higher rates of cGVHD. (30).** |
| *Number and type of previous cell therapies*  *(categorical field, prevcellth)* | **For each cell therapy (alloalloHSCT=1/auto alloHSCT=2/DLI=3/stem cell boost=4) we recommend to capture date (if applicable due to GDPR in EU countries).** | **Recommended** | **This information is a part of the EBMT Med A form. It is mandatory in clinical trials since patients are typically censored at the moment, they receive a new cell product. DLIs contribute to aGVHD and cGVHD (31**)**.** |
| *GVHD prophylaxis*  *(checkboxes for multiple-choice + free field for additional methods if they are not on the list, gvhprop)* | ***Suggested coding categories:***  **PT-Cyclophosphamide**  **CNI**  **MTX**  **MMF**  **mTOR-inhibitor**  **In vivo ATG Depletion**  **Campath-1 depletion**  **Ex vivo αβTCR depletion**  **CD34+ selection**  **Other** | **Mandatory** | **GVHD prophylaxis is a potential risk factor for NRM and predictor of therapy discontinuation (8,9,30).** |

1. **Chronic graft-versus-host disease variables**

**General guidelines for this section:**

**1. All patients not fulfilling the NIH diagnostic criteria should be documented in a special category.**

1. **A categorical field should be created for every organ (values 0-3 (corresponding to non- mild-moderate-severe), as per the 2014 or 2020 NIH cGVHD Staging Criteria. We also recommend using value 4 for ‘organ not assessed’ and value 5 for ˝organ assessed but changes solely due to other causes˝.**
2. **For longitudinal analyses, each follow-up visit should ideally be documented, or at least at day 100, day 180, day 360 and yearly thereafter.**

**The use of EBMT cGVHD application is encouraged for organ scoring (32).**

| *ITEM* | *DEFINITION* | *CATEGORY* | *COMMENTARY* |
| --- | --- | --- | --- |
| *cGVHD*  *(checkboxes,*  *cGVHDtype)* | **cGVHD diagnosed according to the NIH Diagnosis and staging criteria. All items in the NIH Staging Criteria Form should be included.**  **Undefined other cGVHD – atypical signs and symptoms of alloreactivity falling outside the NIH 2014 diagnostic criteria.**  **We recommend that all manifestations treated as cGVHD should be documented. Manifestations possibly connected to cGVHD should be documented, even if they are not treated as such.**  ***Suggested coding categories for the type of cGVHD (multiple checkbox style) :*  „NIH defined cGVHD“=1 (the distinction between 'classic' and 'overlap' is optional) / *˝undefined other cGVHD˝*=2*, as defined by the EBMT-NIH-CIBMTR Taskforce report).*** | **Mandatory** | **NIH cGVHD criteria have been validated and are clinically relevant (33) .**  **2014 NIH criteria use is encouraged (34).**  **Separate fields for onset and type of cGVHD.** |
| *Specific undefined other cGVHD manifestations falling outside of the NIH criteria*  *(checkboxes +free fields, undefother)* | ***Suggested coding categories for type of manifestation (multiple checkbox style)* Immune cytopenias**  **(ITP, hemolysis, agranulocytosis)**  **COP**  **Restrictive allograft syndrome of the lung**  **Cardiac conduction defects**  **Autoimmune pancreatitis**  **Pericardial effusion**  **Pleural effusion**  **Ascites**  **Joint effusion**  **Edema**  **Nephrotic syndrome**  **TMA**  **Myositis**  **Cardiomyopathy**  **Muscle cramps, polyneuropathy, including small fiber involvement**  **CNS manifestations**  **Any auto/alloimmune thyroiditis**  **Pulmonary hypertension**  **Other:**  **Manifestation resulting in change of treatment: yes/no/not available**  ***Suggested coding category: checkbox*** | **Mandatory** | **The majority of these manifestations are described in the diagnosis list of the NIH criteria (35–39).** |
| *Diagnosis of cGVHD*  *(categorical field, gvhdiag)* | ***Suggested coding categories (multiple checkbox style)* Diagnostic criteria present (34).**  **Distinctive symptoms present**  **Biopsy (no evidence of cGVHD=1/possible or likely cGVHD=2/not done=3) Document the specific symptom or sign leading to diagnosis (e.g. free field)** | **Mandatory** | **A significant proportion of patients are treated for distinctive symptoms without histopathological confirmation or other associated symptoms. Therefore, symptoms leading to diagnosis or biopsy should ideally be captured.** |
| *The onset of cGVHD definitions (categorical field, gvhdonset)* | ***Suggested coding categories: De novo=1*– without prior acute GVHD;**  ***Quiescent=2*– after all aGVHD manifestations are resolved or inactive;**  ***progressive=3* – cGVHD onset while aGVHD is still active or unresolved (40)** | **Mandatory** | **Progressive onset is a poor prognostic factor for NRM and OS (41).** |
| *Pulmonary function tests*  *(numerical field for lung function tests values and NIH staging separately, lungnih)* | ***This refers to pulmonary function tests and, if appropriate, imaging performed within 3 months of GVHD diagnosis.***  **Staging is preferentially captured as FEV1 % categories, but if missing, symptom categories can be used but should be marked as based on clinical symptoms only.** | **Mandatory (testing and documentation except children or cases not permitting lung function tests)** | **Staging in the absence of lung function tests should be avoided. FEV1 pre-alloHSCT should be captured for comparison.**  **NIH Lung stage 3 is an adverse survival factor (2005 and 2014) (42).**  **We recommend to add a separate checkbox to mark whether sclerosis or fasciitis of the thoracic wall could contribute to impaired lung function.**  **DLCO should also be captured and corrected for hemoglobin.** |
| *Lung imaging (categorical field, lungimage).* | ***This refers to imaging performed within 3 months of GVHD diagnosis.***  ***Suggested coding categories (multiple checkbox style):***  **evidence of air trapping on HRCT**  **evidence of small airway thickening on HRCT**  **evidence of bronchiectasis on HRCT**  **other signs of GVHD** | **Recommended** |  |
| *Additional Lung PFT (numerical fields).* | **We recommend to document values and change from baseline for: FEV1/VC ratio**  **RV**  **TLC**  **FVC**  **Values after bronchodilator**  **We recommend to document:**  **type of change (obstructive vs restrictive changes)**  **GVHD present on imaging – yes/no/both** | **Recommended** |  |
| *Other lung manifestations and lung disease before alloHSCT if present (numerical field).* | **We recommend to document pre-transplant FEV1, RV, TLC and corrected DLCO** | **Recommended** |  |
| *Liver cGVHD (categorical field for staging, numerical field for the laboratory marker, livernih)* | **Documenting the affected enzymes** | ***Categorical field for staging 0-3 is mandatory.*** | **Hyperbilirubinemia is an adverse OS factor (8). Prognostic value of ALT and AP has not been documented yet.**  **Actual values for liver enzymes and bilirubine need to be recorded to be able to infer response according to NIH 2014 response criteria as well as upper limit of normal (43).** |
| *Skin cGVHD*  *(checkboxes for all manifestations as listed in the 2014 NIH staging criteria, categorical field for staging 0-3, sklinih).* | ***Categorical field for staging 0-3 is mandatory.* Documenting actual body surface area percentage for superficial and deep sclerosis is recommended using the rule of 9's.** | ***Categorical field for staging 0-3 is mandatory.*** |  |
| *Mouth cGVHD (categorical field for staging, mouthnih)* | **Documenting the presence of lichen planus-like features (checkbox - yes/no/not evaluated) is recommended.**  **Separate checkboxes for distinctive (xerostomia, mucocele, mucosal atrophy, pseudomembranes, ulcers) and common (gingivitis, mucositis, erythema, pain) features are recommended.** | ***Categorical field for staging 0-3 is mandatory.* (specific manifestations and symptoms are recommended)** | **Note: OMRS score from NIH 2014 Form A needs to be recorded if response to treatment according to NIH 2014 response criteria (43) is one of the clinical endpoints.** |
| *Eye cGVHD (checkbox for the presence of keratoconjunctivitis sicca and if the patient was examined by an ophthalmologist, categorical field for staging, eyenih)* | **Documenting the presence of keratoconjunctivitis sicca (yes/no/not evaluated) is recommended.**  **Separate checkboxes for distinctive (dry, gritty eyes, cicatricial conjunctivitis, confluent areas of punctate keratopathy) and other (photophobia, periorbital hyperpigmentation, blepharitis) features are recommmended. Documenting whether the patient was examined by an ophthalmologist within 3 months of diagnosis is also recommended** | ***Categorical field for staging 0-3 is mandatory.* (specific signs and symptoms are recommended)** | **Excessive tearing due to inflammation of Meibomian glands should be documented as mild for research purposes.**  **The use of glasses or lenses. or contact lenses to prevent pain or impaired vision due to cGVHD (not the correction of vision) should be documented as severe. The same applies to sunglasses in the absence of sun (inside buildings). As stated in the EBMT-NIH-CIBMTR Taskforce report classifying patients who have received punctal plugs is still an issue.** |
| *Gastrointestinal cGVHD (categorical field for staging, ginih).* | **Special checkboxes for diagnostic (esophageal web, constriction or concentric rings), common features (anorexia, nausea, vomiting, diarrhea, weight loss, and failure to thrive) and exocrine pancreatic insufficiency are recommended. (41)** | ***Categorical field for staging 0-3 is mandatory.* (specific signs and symptoms are recommended)** | **As stated in the EBMT-NIH-CIBMTR Taskforce report classifying patients who have undergone a esophageal dilation or lose a significant amount of weight at first, but stabilize afterward are still unresolved issues.**  **Note: GI scores (esophagus, upper GI and lower GI) from NIH 2014 Form A need to be recorded if response to treatment according to NIH 2014 response criteria (43) is one of the clinical endpoints.** |
| *Joints and Fascia cGVHD (categorical field got staging, jointnih)* | **Special checkboxes for diagnostic (fasciitis), distinctive (myositis, polymyositis) and other features (edema, cramps, arthralgia, muscle weakness, restricted range of motion) are recommended. Documentation of PROM staging is recommended. A checkbox for fasciitis not involving the skin is recommended as well.** | ***Categorical field for staging 0-3 is mandatory.* (specific signs and symptoms are recommended)** | **Note: P-ROM scores from NIH 2014 Form A need to be recorded if response to treatment according to NIH 2014 response criteria (43) is one of the clinical endpoints.** |
| *Genital Tract (categorical field for staging, genitalnih)* | **Women should be examined by a gynecologist within 3 months of cGVHD diagnosis.**  **Special checkboxes for diagnostic and distinctive features are recommended.** | ***Categorical field for staging 0-3 is mandatory.* (specific signs and symptoms are recommended)** | **Lack of a gynecological exam should be captured as well perhaps as a separate checkbox. A special field for males should be included.** |
|  | **Male features: non-infectious balanoposthitis, lichen sclerosis-like or lichen planus-like features, phimosis, or urethra or meatus scarring or stenosis. Document whether or not circumcision was done to alleviate phimosis (checkbox yes/no/not evaluated).** | ***Categorical field for staging 0-3 is mandatory.* (specific signs and symptoms are recommended)** |  |
| *History of* steroid-refractory *acute GVHD (checkbox, yes/no. aGVHD)* | **A history of steroid-refractory aGVHD should be noted (checkbox: yes/no/not evaluated). We recommend documenting previous aGVHD treatment if possible. (free field)** | **Recommended** | **Prior acute GVHD is an adverse factor for NRM and OS (45).** |
| *The maximum grade of prior aGVHD (numerical field, maxacute)* |  | **Recommended** | **Maximum grade of prior aGVHD correlates with NRM and cGVHD risk (1).** |
| *Systemic immunosuppression at the onset of cGVHD (checkbox – yes or no, thsystemic).* | **Glucocorticoid treatment with a dose > 0.5mg/kg/day** | **Mandatory** | **Glucocorticoid treatment with a dose > 0.5mg/kg/day immediately before cGVHD onset is one the most important risk factors for NRM and is regarded as progressive onset type (1,34,46).** |
| *Topical treatment for cGVHD at the time of evaluation (free field. topical).* | **We recommend recording whether topical treatment is used and for which organ.** | **Recommended** | **Topical treatment could be a potential confounder when assessing response to systemic therapy, thus we recommend documenting it for research purposes if available.** |
| *The intensity of systemic immunosuppression at the time of cGVHD evaluation (free field, intensity)* | **Immunosuppression as it has been applied the day before the assessment.** | **Recommended** | **Alternatively, the so-called ˝Sandy´s scale˝ can be used: mild-single agent prednisone up to 0.5 mg/kg/day, moderate-single agent prednisone>0.5 mg/kg/day and/or any single agent modality and high-two or more agents/modalities±prednisone>0.5 mg/kg/day (47).** |
| *cGVHD activity (categorical field, gvhactiv).* | **Clinically active (1)– inflammatory or worsening manifestations regardless of the use of immunosuppressive therapy.**  **Controlled (2)– after all signs of clinical activity have resolved, immunosuppression is ongoing or has been discontinued for less than 24 weeks, regardless of the presence of fixed deficits. If immunosuppression is withheld more than 24 weeks, and no fixed deficits – cGVHD is resolved (3); if stable changes that have not progressed persist after 24 weeks of withheld immunosuppression– cGVHD is considered inactive (4)(40). This classification still needs to be prospectively validated.** | **Recommended** | **Proposed fixed deficits are irreversible scarring manifestations: fibrotic changes, sicca syndrome, as proposed by Cooke et al. (48).** |

| *Steroid refractoriness at the time of evaluation* | Progression of cGVHD A) while on prednisone ≥1 mg/day/kg for one to two weeks, or B) stable cGVHD on ≥0.5 mg/kg/day of prednisone (or 1 mg/kg/day every other day) for one to two months.  Second-line treatment required. | Mandatory | A consensus on whether to use one or two weeks for option A or one or two months for option B is necessary (40).  Steroid refractoriness of cGVHD correlates with NRM (49,50). |
| --- | --- | --- | --- |
| *Steroid dependence at the time of evaluation* | **The inability to control GVHD symptoms while**  **tapering prednisone below 0.25 mg/kg/day (or 0.5 mg/kg every other day) in at least two individual attempts, separated by at least 8 weeks (40).** | **Mandatory** | **Alternatively, the actual dose used in milligrams can be documented.** |
| *Steroid intolerance at evaluation* | **Unacceptable toxicity attributed to steroids.** | **Mandatory** | **All three categories (intolerance, refractoriness, and dependence) should be in a categorical field. Recording side effects is important to characterize the overall burden of disease caused by cGVHD and its treatment (51).** |

| *Clinical manifestations of acute GVHD present at the time of evaluation (currently defined as „overlap GVHD“, checkbox, overlap)* | This type of cGVHD is sometimes referred to as „overlap “category. Clinical signs and symptoms that qualify as „acute “should be documented in detail. The upper and lower gastrointestinal tract should be documented separately. | Mandatory | Poor prognostic factor (34,52,53).  If isolated cholestasis occurs this should be regarded as aGVHD.  Isolated transaminitis without histopathology should not be documented as GVHD and should just be captured as such if cGVHD is not confirmed at another organ site. |
| --- | --- | --- | --- |
| *Lines of therapy at the time of evaluation (numerical for number of previous lines – linenum, free field for the treatment -prevtre)* | **We recommend to record all new treatments started and continued for at least two weeks.** | **Recommended** | **We suggest using a list of agents based on the updated EBMT consensus recommendations (54). Alternatively, a list based on the aforementioned consensus can be made in a checkbox style, with a free field for other, unlisted agents.** |
| *NIH 2014 Global Severity Score (calculated field, nihglobal)* | **Mild – 1 or 2 organ sites - max organ score 1**  **Moderate- 1 organ site scored 2, 3 or more organs scored 1, lung score of 1**  **Severe – score 3 in one or more organs; lung score 2 or 3** | **Mandatory*** | **NIH Global severity score is an adverse NRM and OS factor (55). Lower NIH Global severity is associated with a higher rate of permanent immunosuppression discontinuation (34). * The item should be automatically generated by organ grading using a computer algorithm instead of separate input.** |
| *Platelets < 100000/µl at diagnosis of cGVHD* |  | **Recommended** | **Most relevant risk factor for NRM at diagnosis of cGVHD (56,57). The actual count could be provided as alternative.** |
| *Platelets never reaching 100 000/µl before the onset of cGVHD. (checkbox, yes/no)* |  | **Recommended** | **Thrombocytes <100 000/µl at cGVHD onset are found to be an adverse prognostic factor for NRM and OS in numerous studies but it is currently unclear whether this also applies to patients never engrafted with platelets > 100.000/µl.** |
| *Immunoglobulin levels at the onset of cGVHD (numerical field)* | **IgG, IgA, IgM levels in peripheral blood.** | **Recommended** | **Low immunoglobulin levels post-alloHSCT are associated with decreased overall survival (58), however, the impact of low IgG needs to be studied in cGVHD.** |

1. **Symptom burden, quality of life and functional capacity indicators**

**General comment: When integrating functional test of patient-reported outcomes in routine data collection care should be taken to limit patient burden to a minimum and use the tools as validated. Baseline data is also essential in this context.**

*The tools listed in the fourth part of the Dictionary are non-exclusive examples and many other measures are available.*

| *ITEM* | *DEFINITION* | *CATEGORY* | *COMMENTARY* |
| --- | --- | --- | --- |
| *Lee Symptom Scale* | **A validated cGVHD symptom burden tool designed for longitudinal follow-up.** | **Recommended** | **Useful for capturing symptoms otherwise not reported such as itching (59).** |
| *Short-Form 36* | **Self-assessed functional capacity tool.** | **Recommended** | **Used in cGVHD population functional assessment (47).** |
| *Human Activity Profile* | **Self-assessment tool evaluating physical activity and energy expenditure.** | **Recommended** | **Validated in cGVHD/post-alloHSCT patient populations (60,61). Potential measure for frailty assessment in cGVHD (62)(63).** |
| *Patient NIH form B/clinician assessed NIH form B* | **Self-assessment tool by which the patient or health care provider assess response.** | **Recommended** | **These measures strongly correlate with overall survival and failure-free survival (64).** |
| *2-minute walk test or 6-minute walk test* | **A measure of functional capacity in chronically ill patients** | **Recommended** | **Validated for use in cGVHD (47,65).** |
| *Upper and lower limb range of motion (PROM)* | **A measure of functional capacity.** | **Recommended** | **An objective measure of the joint range of motion used in cGVHD (47).** |

**Mandatory items are necessary for a database/registry and are fundamental pieces of information, which should be considered for data analysis. This recommendation is evidence-based with regard to the impact of relevant endpoints in cGVHD research. Recommended items are those not essential for a database, but can add value to research and should be included if they are available and feasible or required for specific endpoints.**

**Abbreviations:**

**UPN – unique patient number**

**EBMT – European Society for Blood and Marrow Transplantation**

**CIBMTR – Center for International Blood and Marrow Transplant Research**

**cGVHD – chronic graft versus host disease**

**alloHSCT – allogeneic hematopoietic stem cell transplantation**

**KPS – Karnofsky performance status**

**NRM – non-relapse mortality**

**OS – overall survival**

**MRD – minimal residual disease**

**TBI – total body irradiation**

**Gy – Gray**

**Bu – busulfan**

**Flu – fludarabine**

**Cy – cyclophoshpamide**

**Ida – idarubicin**

**AraC – Cytarabine**

**ATG – antithymocyte globulin**

**CMV – cytomegalovirus**

**TRM – transplant-related mortality**

**HLA – human leukocyte antigen**

**PBSC – peripheral blood stem cells**

**G-CSF – granulocyte colony-stimulating factor**

**CNI – calcineurin inhibitor**

**MTX – methotrexate**

**ITP – immune thrombocytopenia**

**COP – Cryptogenic organizing pneumonia**

**CNS – central nervous system**

**HRCT – high resolution computerized tomography**

**FEV1% – forced expiratory volume in the 1^st^second percentage**

**RV – residual volume**

**TLC – total lung capacity**

**DLCO – diffusing capacity for carbon monoxide**

**CTCAE – Common Terminology Criteria for Adverse Events**

**PROM – photographic range of motion**

**FACT-BMT – Functional Assessment of Cancer Therapy -Bone Marrow Transplantation**

**PT – post-transplantation**

**TMA – thrombotic microangiopathy**

**References:**

1. Stewart BL, Storer B, Storek J, Deeg HJ, Storb R, Hansen JA, et al. Duration of immunosuppressive treatment for chronic graft-versus-host disease. Blood [Internet]. 2004 Dec 1 [cited 2019 Jan 23];104(12):3501–6. Available from: http://www.bloodjournal.org/cgi/doi/10.1182/blood-2004-01-0200

2. Kondo M, Kojima S, Horibe K, Kato K, Matsuyama T. Risk factors for chronic graft-versus-host disease after allogeneic stem cell transplantation in children. Bone Marrow Transplant [Internet]. 2001 Apr 4 [cited 2019 Jan 24];27(7):727–30. Available from: http://www.ncbi.nlm.nih.gov/pubmed/11360113

3. Madbouly A, Wang T, Haagenson M, Paunic V, Vierra-Green C, Fleischhauer K, et al. Investigating the Association of Genetic Admixture and Donor/Recipient Genetic Disparity with Transplant Outcomes. Biol Blood Marrow Transplant [Internet]. 2017 Jun [cited 2019 Feb 26];23(6):1029–37. Available from: http://www.ncbi.nlm.nih.gov/pubmed/28263917

4. Ford ME, Kelly PA. Conceptualizing and categorizing race and ethnicity in health services research. Health Serv Res [Internet]. 2005 Oct [cited 2019 Feb 26];40(5 Pt 2):1658–75. Available from: http://www.ncbi.nlm.nih.gov/pubmed/16179001

5. Spierings E, Hendriks M, Absi L, Canossi A, Chhaya S, Crowley J, et al. Phenotype frequencies of autosomal minor histocompatibility antigens display significant differences among populations. PLoS Genet [Internet]. 2007 Jun [cited 2019 Feb 26];3(6):e103. Available from: http://www.ncbi.nlm.nih.gov/pubmed/17604453

6. Lee SJ, Klein JP, Barrett AJ, Ringden O, Antin JH, Cahn J-Y, et al. Severity of chronic graft-versus-host disease: association with treatment-related mortality and relapse. Blood [Internet]. 2002 Jul 15 [cited 2019 Jan 31];100(2):406–14. Available from: http://www.ncbi.nlm.nih.gov/pubmed/12091329

7. Arora M, Hemmer MT, Ahn KW, Klein JP, Cutler CS, Urbano-Ispizua A, et al. Center for International Blood and Marrow Transplant Research chronic graft-versus-host disease risk score predicts mortality in an independent validation cohort. Biol Blood Marrow Transplant [Internet]. 2015 Apr [cited 2019 Jan 30];21(4):640–5. Available from: http://www.ncbi.nlm.nih.gov/pubmed/25528390

8. Arora M, Klein JP, Weisdorf DJ, Hassebroek A, Flowers MED, Cutler CS, et al. Chronic GVHD risk score: a Center for International Blood and Marrow Transplant Research analysis. Blood [Internet]. 2011 Jun 16 [cited 2019 Jan 30];117(24):6714–20. Available from: http://www.ncbi.nlm.nih.gov/pubmed/21493797

9. Inamoto Y, Kim DD, Storer BE, Moon JH, Lipton JH, Kuruvilla J, et al. Application of CIBMTR risk score to NIH chronic GVHD at individual centers. Blood [Internet]. 2014 Jan 16 [cited 2019 Jan 30];123(3):453–5. Available from: http://www.ncbi.nlm.nih.gov/pubmed/24435000

10. Gratwohl A. The EBMT risk score. Bone Marrow Transplant [Internet]. 2012 Jun 6 [cited 2019 Feb 3];47(6):749–56. Available from: http://www.ncbi.nlm.nih.gov/pubmed/21643021

11. Armand P, Kim HT, Logan BR, Wang Z, Alyea EP, Kalaycio ME, et al. Validation and refinement of the Disease Risk Index for allogeneic stem cell transplantation. Blood [Internet]. 2014 Jun 5 [cited 2019 Sep 26];123(23):3664–71. Available from: http://www.ncbi.nlm.nih.gov/pubmed/24744269

12. Jacobsohn DA, Arora M, Klein JP, Hassebroek A, Flowers ME, Cutler CS, et al. Risk factors associated with increased nonrelapse mortality and with poor overall survival in children with chronic graft-versus-host disease. Blood [Internet]. 2011 Oct 20 [cited 2019 Jan 30];118(16):4472–9. Available from: http://www.ncbi.nlm.nih.gov/pubmed/21878671

13. Bacigalupo A, Ballen K, Rizzo D, Giralt S, Lazarus H, Ho V, et al. DEFINING THE INTENSITY OF CONDITIONING REGIMENS : working definitions. Biol Blood Marrow Transplant [Internet]. 2009 [cited 2019 Apr 3];15(12):1628. Available from: https://www.ncbi.nlm.nih.gov/pmc/articles/PMC2861656/

14. Couriel DR, Saliba RM, Giralt S, Khouri I, Andersson B, de Lima M, et al. Acute and chronic graft-versus-host disease after ablative and nonmyeloablative conditioning for allogeneic hematopoietic transplantation. Biol Blood Marrow Transplant [Internet]. 2004 Mar 1 [cited 2019 Jan 24];10(3):178–85. Available from: https://www.sciencedirect.com/science/article/pii/S1083879103004191

15. Giralt S, Ballen K, Rizzo D, Bacigalupo A, Horowitz M, Pasquini M, et al. Reduced-intensity conditioning regimen workshop: defining the dose spectrum. Report of a workshop convened by the center for international blood and marrow transplant research. Biol Blood Marrow Transplant [Internet]. 2009 Mar [cited 2019 Apr 3];15(3):367–9. Available from: http://www.ncbi.nlm.nih.gov/pubmed/19203728

16. Martires KJ, Baird K, Steinberg SM, Grkovic L, Joe GO, Williams KM, et al. Sclerotic-type chronic GVHD of the skin: clinical risk factors, laboratory markers, and burden of disease. Blood [Internet]. 2011 Oct 13 [cited 2019 Jan 24];118(15):4250–7. Available from: http://www.ncbi.nlm.nih.gov/pubmed/21791415

17. Kollman C, Howe CWS, Anasetti C, Antin JH, Davies SM, Filipovich AH, et al. Donor characteristics as risk factors in recipients after transplantation of bone marrow from unrelated donors: the effect of donor age. Blood. 2001 Oct 1;98(7):2043–51.

18. Paz A, Rigoni L, Fischer G, Schittler M, Pezzi A, Valim V, et al. Donor characteristics and hematopoietic stem cell transplantation outcome: experience of a single center in Southern Brazil. Hematol Transfus Cell Ther [Internet]. 2018 Apr [cited 2019 Jan 31];40(2):136–42. Available from: http://www.ncbi.nlm.nih.gov/pubmed/30057987

19. George B, Pati N, Gilroy N, Ratnamohan M, Huang G, Kerridge I, et al. Pre-transplant cytomegalovirus (CMV) serostatus remains the most important determinant of CMV reactivation after allogeneic hematopoietic stem cell transplantation in the era of surveillance and preemptive therapy. Transpl Infect Dis [Internet]. 2010 Aug 1 [cited 2019 Jan 30];12(4):322–9. Available from: http://www.ncbi.nlm.nih.gov/pubmed/20487414

20. Kalra A, Williamson T, Daly A, Savoie ML, Stewart DA, Khan F, et al. Impact of Donor and Recipient Cytomegalovirus Serostatus on Outcomes of Antithymocyte GlobulineConditioned Hematopoietic Cell Transplantation. Biol Blood Marrow Transplant [Internet]. 2016 [cited 2021 Mar 14];22:1654–63. Available from: http://dx.doi.org/10.1016/j.bbmt.2016.05.020

21. Jaskula E, Dlubek D, Tarnowska A, Lange J, Mordak-Domagala M, Suchnicki K, et al. Anti-CMV-IgG Positivity of Donors Is Beneficial for alloHSCT Recipients with Respect to the Better Short-Term Immunological Recovery and High Level of CD4+CD25high Lymphocytes. Viruses [Internet]. 2015 Mar 23 [cited 2019 Jan 30];7(3):1391–408. Available from: http://www.ncbi.nlm.nih.gov/pubmed/25807050

22. Ljungman P, Brand R, Einsele H, Frassoni F, Niederwieser D, Cordonnier C. Donor CMV serologic status and outcome of CMV-seropositive recipients after unrelated donor stem cell transplantation: An EBMT megafile analysis. Blood. 2003 Dec 15;102(13):4255–60.

23. Q46-83: Donor Information - Forms Instruction Manual - 1 [Internet]. [cited 2020 May 15]. Available from: https://www.cibmtr.org/manuals/fim/1/en/topic/donor-information

24. Storb R, Prentice RL, Sullivan KM, Shulman HM, Deeg HJ, Doney KC, et al. Predictive factors in chronic graft-versus-host disease in patients with aplastic anemia treated by marrow transplantation from HLA-identical siblings. Ann Intern Med. 1983;98(4):461–6.

25. Carlens S, Ringdén O, Remberger M, Lönnqvist B, Hägglund H, Klaesson S, et al. Risk factors for chronic graft-versus-host disease after bone marrow transplantation: A retrospective single centre analysis. Bone Marrow Transplant. 1998;22(8):755–61.

26. Remberger M, Kumlien G, Aschan J, Barkholt L, Hentschke P, Ljungman P, et al. Risk factors for moderate-to-severe chronic graft-versus-host disease after allogeneic hematopoietic stem cell transplantation. Biol Blood Marrow Transplant [Internet]. 2002 [cited 2019 Jan 31];8(12):674–82. Available from: http://www.ncbi.nlm.nih.gov/pubmed/12523580

27. Tekgündüz SA, Özbek N. ABO blood group mismatched hematopoietic stem cell transplantation. Vol. 54, Transfusion and Apheresis Science. Elsevier Ltd; 2016. p. 24–9.

28. Storek J, Dawson MA, Storer B, Stevens-Ayers T, Maloney DG, Marr KA, et al. Immune reconstitution after allogeneic marrow transplantation compared with blood stem cell transplantation. Blood. 2001 Jun 1;97(11):3380–9.

29. Holtick U, Albrecht M, Chemnitz JM, Theurich S, Skoetz N, Scheid C, et al. Bone marrow versus peripheral blood allogeneic haematopoietic stem cell transplantation for haematological malignancies in adults. Cochrane Database Syst Rev [Internet]. 2014 Apr 20 [cited 2019 Jan 30];(4):CD010189. Available from: http://www.ncbi.nlm.nih.gov/pubmed/24748537

30. Curtis LM, Pirsl F, Steinberg SM, Mitchell SA, Baird K, Cowen EW, et al. Predictors for Permanent Discontinuation of Systemic Immunosuppression in Severely Affected Chronic Graft-Versus-Host Disease Patients. Biol Blood Marrow Transplant [Internet]. 2017 Nov 1 [cited 2018 Jan 25];23(11):1980–8. Available from: http://www.ncbi.nlm.nih.gov/pubmed/28797782

31. Frey N V, Porter DL. Graft-versus-host disease after donor leukocyte infusions: presentation and management. Best Pract Res Clin Haematol [Internet]. 2008 Jun [cited 2019 Jan 30];21(2):205–22. Available from: http://www.ncbi.nlm.nih.gov/pubmed/18503987

32. Schoemans HM, Goris K, Van Durm R, Fieuws S, De Geest S, Pavletic SZ, et al. The eGVHD app has the potential to improve the accuracy of graft-versus-host disease assessment: A multicenter randomized controlled trial. Haematologica. 2018 Sep 30;103(10):1698–707.

33. Baird K, Steinberg SM, Grkovic L, Pulanic D, Cowen EW, Mitchell SA, et al. National Institutes of Health Chronic Graft-versus-Host Disease Staging in Severely Affected Patients: Organ and Global Scoring Correlate with Established Indicators of Disease Severity and Prognosis. Biol Blood Marrow Transplant [Internet]. 2013 Apr [cited 2017 Sep 7];19(4):632–9. Available from: http://linkinghub.elsevier.com/retrieve/pii/S1083879113000372

34. Jagasia MH, Greinix HT, Arora M, Williams KM, Wolff D, Cowen EW, et al. National Institutes of Health Consensus Development Project on Criteria for Clinical Trials in Chronic Graft-versus-Host Disease: I. The 2014 Diagnosis and Staging Working Group Report. Biol Blood Marrow Transplant [Internet]. 2015 Mar [cited 2017 Sep 7];21(3):389-401.e1. Available from: http://www.ncbi.nlm.nih.gov/pubmed/25529383

35. Grauer O, Wolff D, Bertz H, Greinix H, Kuhl J-S, Lawitschka A, et al. Neurological manifestations of chronic graft-versus-host disease after allogeneic haematopoietic stem cell transplantation: report from the Consensus Conference on Clinical Practice in chronic graft-versus-host disease. Brain [Internet]. 2010 Oct 1 [cited 2019 Sep 30];133(10):2852–65. Available from: http://www.ncbi.nlm.nih.gov/pubmed/20846944

36. Kraus PD, Wolff D, Grauer O, Angstwurm K, Jarius S, Wandinger KP, et al. Muscle Cramps and Neuropathies in Patients with Allogeneic Hematopoietic Stem Cell Transplantation and Graft-versus-Host Disease. PLoS One. 2012 Sep 17;7(9).

37. Leonard JT, Newell LF, Meyers G, Hayes-Lattin B, Gajewski J, Heitner S, et al. Chronic GvHD-associated serositis and pericarditis. Bone Marrow Transplant [Internet]. 2015 Aug 11 [cited 2019 Sep 30];50(8):1098–104. Available from: http://www.ncbi.nlm.nih.gov/pubmed/25961774

38. Karakulska-Prystupiuk E, Basak G, Dwilewicz-Trojaczek J, Paluszewska M, Boguradzki P, Jędrzejczak W. Pericarditis in Patients With Chronic Graft-vs-Host Disease. Transplant Proc [Internet]. 2018 Sep [cited 2019 Sep 30];50(7):2218–22. Available from: http://www.ncbi.nlm.nih.gov/pubmed/30177139

39. Hu SL. The role of graft-versus-host disease in haematopoietic cell transplantation-associated glomerular disease. Nephrol Dial Transplant [Internet]. 2011 Jun [cited 2019 Sep 30];26(6):2025–31. Available from: http://www.ncbi.nlm.nih.gov/pubmed/20961888

40. Schoemans HM, Lee SJ, Ferrara JL, Wolff D, Levine JE, Schultz KR, et al. EBMT−NIH−CIBMTR Task Force position statement on standardized terminology &amp; guidance for graft-versus-host disease assessment. Bone Marrow Transplant [Internet]. 2018 Nov 5 [cited 2019 Jan 31];53(11):1401–15. Available from: http://www.ncbi.nlm.nih.gov/pubmed/29872128

41. Grube M, Holler E, Weber D, Holler B, Herr W, Wolff D. Risk Factors and Outcome of Chronic Graft-versus-Host Disease after Allogeneic Stem Cell Transplantation—Results from a Single-Center Observational Study. Biol Blood Marrow Transplant [Internet]. 2016 Oct [cited 2019 Jan 31];22(10):1781–91. Available from: http://www.ncbi.nlm.nih.gov/pubmed/27343720

42. Kerep AZ, Broome J, Pirsl F, Curtis LM, Steinberg SM, Mitchell SA, et al. Impact of the 2014 NIH chronic graft-versus-host disease scoring criteria modifications assessed in a large cohort of severely affected patients. Bone Marrow Transplant [Internet]. 2018 Aug; Available from: http://europepmc.org/abstract/med/30089900

43. Lee SJ, Wolff D, Kitko C, Koreth J, Inamoto Y, Jagasia M, et al. Measuring Therapeutic Response in Chronic Graft-versus-Host Disease. National Institutes of Health Consensus Development Project on Criteria for Clinical Trials in Chronic Graft-versus-Host Disease: IV. The 2014 Response Criteria Working Group Report. Biol Blood Marrow Transplant. 2015 Jun 1;21(6):984–99.

44. Nakasone H, Ito A, Endo H, Kida M, Koji I, Usuki K. Pancreatic atrophy is associated with gastrointestinal chronic GVHD following allogeneic PBSC transplantation. Bone Marrow Transplant [Internet]. 2010 Mar 20 [cited 2019 Sep 29];45(3):590–2. Available from: http://www.ncbi.nlm.nih.gov/pubmed/19617903

45. Ma CKK, García-Cadenas I, Fox ML, Ai S, Nivison-Smith I, Milliken ST, et al. Poor prognosis in patients with steroid refractory acute graft versus host disease treated with etanercept: a multi-centre analysis [Internet]. Vol. 53, Bone Marrow Transplantation. Nature Publishing Group; 2018 [cited 2021 Mar 15]. p. 1478–82. Available from: https://doi.org/10.1038/s41409-018-0215-4

46. Vigorito AC, Campregher P V, Storer BE, Carpenter PA, Moravec CK, Kiem H-P, et al. Evaluation of NIH consensus criteria for classification of late acute and chronic GVHD. Blood [Internet]. 2009 Jul 16 [cited 2019 Jan 31];114(3):702–8. Available from: http://www.ncbi.nlm.nih.gov/pubmed/19470693

47. Mitchell SA, Leidy NK, Mooney KH, Dudley WN, Beck SL, LaStayo PC, et al. Determinants of functional performance in long-term survivors of allogeneic hematopoietic stem cell transplantation with chronic graft-versus-host disease (cGVHD). Bone Marrow Transplant [Internet]. 2010 Apr 28 [cited 2017 Sep 7];45(4):762–9. Available from: http://www.nature.com/doifinder/10.1038/bmt.2009.238

48. Cooke KR, Luznik L, Sarantopoulos S, Hakim FT, Jagasia M, Fowler DH, et al. The Biology of Chronic Graft-versus-Host Disease: A Task Force Report from the National Institutes of Health Consensus Development Project on Criteria for Clinical Trials in Chronic Graft-versus-Host Disease. Biol Blood Marrow Transplant [Internet]. 2017 Feb [cited 2017 Sep 23];23(2):211–34. Available from: http://www.ncbi.nlm.nih.gov/pubmed/27713092

49. Miklos D, Cutler CS, Arora M, Waller EK, Jagasia M, Pusic I, et al. Ibrutinib for chronic graft-versus-host disease after failure of prior therapy. Blood [Internet]. 2017 Jan 23 [cited 2018 Jan 27];130(21):2243–50. Available from: http://www.ncbi.nlm.nih.gov/pubmed/28924018

50. Wolff D, Gerbitz A, Ayuk F, Kiani A, Hildebrandt GC, Vogelsang GB, et al. Consensus Conference on Clinical Practice in Chronic Graft-versus-Host Disease (GVHD): First-Line and Topical Treatment of Chronic GVHD. Biol Blood Marrow Transplant [Internet]. 2010 Dec [cited 2019 Jan 31];16(12):1611–28. Available from: http://www.ncbi.nlm.nih.gov/pubmed/20601036

51. Wolff D, Schleuning M, von Harsdorf S, Bacher U, Gerbitz A, Stadler M, et al. Consensus Conference on Clinical Practice in Chronic GVHD: Second-Line Treatment of Chronic Graft-versus-Host Disease. Biol Blood Marrow Transplant [Internet]. 2011 Jan [cited 2019 Jan 31];17(1):1–17. Available from: http://www.ncbi.nlm.nih.gov/pubmed/20685255

52. Filipovich AH, Weisdorf D, Pavletic S, Socie G, Wingard JR, Lee SJ, et al. National Institutes of Health consensus development project on criteria for clinical trials in chronic graft-versus-host disease: I. Diagnosis and staging working group report. Biol Blood Marrow Transplant [Internet]. 2005 Dec 1 [cited 2017 Sep 7];11(12):945–56. Available from: http://www.ncbi.nlm.nih.gov/pubmed/16338616

53. Lee SJ. Classification systems for chronic graft-versus-host disease. Blood [Internet]. 2017 Jan 5 [cited 2017 Sep 7];129(1):30–7. Available from: http://www.ncbi.nlm.nih.gov/pubmed/27821503

54. Penack O, Marchetti M, Ruutu T, Aljurf M, Bacigalupo A, Bonifazi F, et al. Prophylaxis and management of graft versus host disease after stem-cell transplantation for haematological malignancies: updated consensus recommendations of the European Society for Blood and Marrow Transplantation [Internet]. Vol. 7, The Lancet Haematology. Elsevier Ltd; 2020 [cited 2021 Mar 15]. p. e157–67. Available from: https://pubmed.ncbi.nlm.nih.gov/32004485/

55. Pérez-Simón JA, Afram G, Martino R, Piñana JL, Caballero-Velazquez T, Ringden O, et al. Evaluation of prognostic factors among patients with chronic graft-versus-host disease. Haematologica [Internet]. 2012 Aug 1 [cited 2019 Feb 1];97(8):1187–95. Available from: http://www.haematologica.org/cgi/doi/10.3324/haematol.2011.055244

56. Pulanic D, Lozier JN, Pavletic SZ. Thrombocytopenia and hemostatic disorders in chronic graft versus host disease. Bone Marrow Transplant [Internet]. 2009 Oct 17 [cited 2019 Feb 1];44(7):393–403. Available from: http://www.nature.com/articles/bmt2009196

57. Pavletic SZ, Smith LM, Bishop MR, Lynch JC, Tarantolo SR, Vose JM, et al. Prognostic factors of chronic graft-versus-host disease after allogeneic blood stem-cell transplantation. Am J Hematol [Internet]. 2005 Apr [cited 2019 Jan 30];78(4):265–74. Available from: http://doi.wiley.com/10.1002/ajh.20275

58. Norlin AC, Sairafi D, Mattsson J, Ljungman P, Ringdén O, Remberger M. Allogeneic stem cell transplantation: Low immunoglobulin levels associated with decreased survival. Bone Marrow Transplant. 2008 Feb;41(3):267–73.

59. Lee S k, Cook EF, Soiffer R, Antin JH. Development and validation of a scale to measure symptoms of chronic graft-versus-host disease. Biol Blood Marrow Transplant [Internet]. 2002 [cited 2017 Sep 7];8(8):444–52. Available from: http://www.ncbi.nlm.nih.gov/pubmed/12234170

60. Herzberg PY, Heussner P, Mumm FHA, Horak M, Hilgendorf I, von Harsdorf S, et al. Validation of the Human Activity Profile Questionnaire in Patients after Allogeneic Hematopoietic Stem Cell Transplantation. Biol Blood Marrow Transplant [Internet]. 2010 Dec [cited 2017 Sep 7];16(12):1707–17. Available from: http://linkinghub.elsevier.com/retrieve/pii/S1083879110002430

61. Davidson M, de Morton N. A systematic review of the Human Activity Profile. Clin Rehabil [Internet]. 2007 Feb 1 [cited 2017 Sep 9];21(2):151–62. Available from: http://journals.sagepub.com/doi/10.1177/0269215506069475

62. Arora M, Sun C-L, Ness KK, Teh JB, Wu J, Francisco L, et al. Physiologic Frailty in Nonelderly Hematopoietic Cell Transplantation Patients. JAMA Oncol [Internet]. 2016 Oct 1 [cited 2018 Feb 15];2(10):1277. Available from: http://www.ncbi.nlm.nih.gov/pubmed/27254472

63. Buta BJ, Walston JD, Godino JG, Park M, Kalyani RR, Xue Q-L, et al. Frailty assessment instruments: Systematic characterization of the uses and contexts of highly-cited instruments. Ageing Res Rev [Internet]. 2016 Mar 1 [cited 2019 Jan 18];26:53–61. Available from: https://www.sciencedirect.com/science/article/pii/S1568163715300386?via%3Dihub

64. Palmer J, Chai X, Pidala J, Inamoto Y, Martin PJ, Storer B, et al. Predictors of survival, nonrelapse mortality, and failure-free survival in patients treated for chronic graft-versus-host disease. Blood [Internet]. 2016 Jan 7 [cited 2019 Sep 30];127(1):160–6. Available from: http://www.ncbi.nlm.nih.gov/pubmed/26527676

65. Pidala J, Chai X, Martin P, Inamoto Y, Cutler C, Palmer J, et al. Hand grip strength and 2-minute walk test in chronic graft-versus-host disease assessment: analysis from the Chronic GVHD Consortium. Biol Blood Marrow Transplant [Internet]. 2013 Jun [cited 2017 Sep 7];19(6):967–72. Available from: http://linkinghub.elsevier.com/retrieve/pii/S1083879113001407

Module that could be used in research studies and registries to document the presence of atypical manifestations of chronic GVHD, including provisional definitions (Cuvelier G, Schoettler M, Buxbaum N, Pinal-Fernandez I, Schmalzing M, Distler J, et al. **(In press)** Towards a Better Understanding of the Atypical Features of Chronic Graft-Versus-Host Disease: A Report from the 2020 National Institutes of Health Consensus Project Task Force. Transplant Cell Ther. 2022;)

| **Atypical manifestation of GVHD** | **Provisional Definition** | **Present (mark 🗴)** |
| --- | --- | --- |
| 1. **Immune mediated cytopenias** |  |  |
| Immune-mediated neutropenia | Neutrophils < 0.5 x 10^9^/L after primary engraftment not caused by toxicity or infection; the presence of anti-neutrophil antibodies should be reported, but is not essential for diagnosis.^1^ |  |
| Hemolytic anemia | Antibody mediated hemolytic anemia with a drop of the hemoglobin level excluding patients with pure red cell aplasia/hemolysis due to major ABO incompatible donor and hemolysis due to TAM^2^ |  |
| Immune mediated thrombocytopenia | Platelets < 50 x 10^9^/L with an acute or subacute decline ≥50 x 10^9^/L from previous values and signs of high turn over and exclusion of toxicity, infection, transplant associated thrombotic microangiopathy, or disseminated intravascular coagulation; the presence of anti-platelet antibodies should be reported but is not essential for diagnosis^1^ |  |
| Evans syndrome | Combination of immune mediated hemolysis and thrombocytopenia and / or neutropenia |  |
| Transplant associated microangiopathia (TAM) | Anti-erythrocytic antibody negative hemolysis with schistocytes and de novo or prolonged thrombocytopenia (<50/nl; over 50% or greater reduction from previous counts); sudden and persistent increase in lactate dehydrogenase concentration; decrease in hemoglobin concentration or increased transfusion requirement; and decrease in serum haptoglobin^3^ |  |
| **2. GASTROINTESTINAL** |  |  |
| Immune-mediated pancreatitis | Pancreatitis not caused by biliary obstruction or other identifiable causes, responding to immunosuppression (corticosteroids)^4^ |  |
| **3. PULMONARY** |  |  |
| Organizing pneumonia | Restrictive impairment with reduced TLC with FEV_1_/FVC > LLN with patchy and peribronchial infiltrates or consolidation, and reticular ground glass opacities, responding to corticosteroids^5, 6^ |  |
| Non-specific interstitial pneumonia | Reduced TLC and DLCO including a decrease of FEV1 > 10% from baseline and a FEV1/FVC > 0.7 excluding extrapulmonary and infectious causes with confluent bilateral lower lobe ground glass opacities, bronchiectasis and lower lobe volumes loss^6-9^ |  |
| Pleuroparenchymal pulmonary fibroelastosis | Reduced TLC and DLCO, including a decrease of FEV1 > 10% from baseline and a FEV1/FVC > 0.7 excluding extrapulmonary and infectious cause, upper lobe fibrosis with subpleural and pleural thickening, loss of lung volume, and lower lobe traction bronchiectasis^7, 10-13^ |  |
| **4. ENDOCRINE** |  |  |
| Thyroiditis - Hashimoto´s disease | Thyroiditis with detection of thyroid peroxidase (TPO) auto-antibodies |  |
| Thyroiditis - Graves´disease | Thyroiditis with detection of thyroid stimulating immunoglobulin (TSI) auto-antibodies |  |
| **5. CENTRAL NERVOUS SYSTEM** |  |  |
| Neurocognitive Deficits | Neurocognitive deficits starting or progressing post 3 months after alloSCT not fully explained by medication or infections |  |
| Meningoencephalitis | Meningoencephalitis starting 3 months or greater after transplantation; not caused by infection or medicatation; associated frequently with polyclonal cerebrospinal fluid lymphocytosis ^14^ |  |
| Multiple Sclerosis-like Encephalitis | Patchy leukoencephalopathy, optic nerve and/or spinal cord involvement not caused by infections or medication, associated frequently with polyclonal cerebrospinal fluid lymphocytosis, cerebrospinal fluid specific oligoclonal IgG bands supportive; including neuromyelitis optica spectrum disease like courses characterised by autoantibodies against aquaporin 4, myelin-oligodendrocyte glycoprotein (MOG) and/or glial fibrillary acidic protein (GFAP).^15^ |  |
| CNS vasculitis-like disorders | Vascular CNS events, radiological signs of local ischemia / stroke not explained by other vascular diseases like embolism or arteriosclerosis, infections or medication ^16, 17^ |  |
| **6. PERIPHERAL NERVOUS SYSTEM** |  |  |
| Chronic Inflammatory Demyelinating Polyneuropathy (CIDP) | Progressive motor and/or sensory loss progressing for more than 8 weeks, demyelinating polyneuropathy in neurophysiology (reduced nerve conduction studies, conduction blocks in nerve conduction studies, prolongation of F-wave latency) not caused by infections or medication, elevated protein level in cerebrospinal fluid without cell count >20/µl and gadolinium enhancement of nerve roots in MRI as supportive criteria, diagnostic criteria for CIDP may not be met completely^14, 18, 19^ |  |
| Guillain-Barré syndrome (GBS) | Progressive motor and/or sensory loss, loss of deep tendon reflexes and possible autonomic dysfunction progressing for less than 6 weeks, demyelinating polyneuropathy in neurophysiology (reduced nerve conduction studies, conduction blocks in nerve conduction studies, prolongation of F-wave latency) not caused by infections or medication, elevated protein level in cerebrospinal fluid without cell count >20/µl as supportive criteria, diagnostic criteria for GBS may not be met completely^20, 21^ |  |
| Small Fiber Polyneuropathy (SFN) | Progressive signs and symptoms not caused by infections or medication involving autonomic or sensory SFN.  Autonomic SFN: diarrhea/obstipation, dry mouth/eyes, other  Sensory SFN: neuropathic pain, elevated threshold for pain, warm and cold sensation, positive sensory signs^22^ |  |
| Myasthenia gravis | Varying degrees of painless skeletal muscle weakness affecting eyes, face, swallowing and extremities pronounced in the evening, decreasing strength under repetition, decrease of amplitudes in repetitive nerve stimulation, improvement after cholinesterase inhibitors like pyridostigmin, supportive: autoantibodies against Acethylcholin receptor and/or muscle specific kinase (MuSK) (caveat: autoantibodies frequently positive without signs of myasthenia gravis).^23, 24^ |  |
| Other peripheral neuropathies | Multifocal motor neuropathy not explained by toxicity or infection |  |
| **7. RENAL** |  |  |
| Macroalbuminuria, Nephrotic-range proteinuria, unexplained significant increase in proteinuria | Macroalbuminuria, nephrotic-range proteinuria or significant increase in proteinuria (at least >0.5g/24h) not caused by pre-existing diseases of the glomerular filtration barrier (e.g. hereditary proteinuria, diabetic nephropathy or pre-existing glomerulonephritis)^25^ |  |
| Glomerular (various types of glomerulonephritis) and tubulointerstitial damage | Different pathologies of intrarenal compartments not caused by prerenal/postrenal damage or pre-existing renoparenchymal disease^26^ |  |
| Renal thrombotic microangiopathy | Renal insufficiency due to unexplained thrombotic microangiopathy (either as part of systemic TAM or limited to the kidneys. Isolated renal thrombotic microangioathy usually requires histopathological confirmation).^26^ Exclusion of other causes of TMA including medications and radiation. |  |
| **8. MUSCLES, FASCIA, JOINTS** |  |  |
| Edema | Edema not explained by volume overload, hypo-albuminemia, side effects of medication or vascular damage (thrombosis, venous insufficiency) |  |
| Muscle cramps | Sudden and involuntary, often painful, contraction of one or more muscle groups not solely explained by magnesium deficiency or side effects of medication, and interfering significantly with activities of daily living^27^ |  |
| Arthralgia | Joint pain or articular tenderness excluding non-immunolgical-related causes (e.g. degenerative joint diseases) |  |
| Arthritis | Arthralgia caused by objectified inflammation of the articular synovium |  |
| Myositis | Muscle weakness, elevated muscle enzymes, and/or myopathic changes on biopsy, electromyography, or MRI^28^ |  |
| **9. OTHER** |  |  |
| Cardiac conduction abnormalities | Cardiac conduction abnormalities not explained by ischemic damage, medication or infectious causes ^29^ |  |
| Cardiomyopathy/Myocarditis | Cardiomyopathy/myocarditis not explained by ischemic damage, toxicity or infectious causes ^30^ |  |
| Vasculitis | Inflammation of blood vessel wall objectified by biopsy (for small blood vessels, mainly skin, kidney), angiography (for medium-sized and large vessels) or other imaging (for large vessels) |  |
| Serositis - pericardial and pleural effusions, ascites | Effusions not explained by volume overload, side effects of medication (e.g., tyrosine kinase inhibitors), infections or relapse of the underlying malignancy ^31^ |  |
| Raynaud’s phenomenon | Transient reversible ischemia of fingers or toes always starting with sharply demarcated pallor of at least one digit, possibly followed by cyanotic skin of the involved area, and possibly hyperemic redness upon reperfusion |  |

**Please list any suspected atypical manifestation of chronic GVHD which is present but not captured above:**

__________________________________________________________________________
